# The effect of lysophosphatidic acid during *in vitro* maturation of bovine cumulus–oocyte complexes: cumulus expansion, glucose metabolism and expression of genes involved in the ovulatory cascade, oocyte and blastocyst competence

**DOI:** 10.1186/s12958-015-0044-x

**Published:** 2015-05-16

**Authors:** Dorota Boruszewska, Emilia Sinderewicz, Ilona Kowalczyk-Zieba, Katarzyna Grycmacher, Izabela Woclawek-Potocka

**Affiliations:** Department of Reproductive Immunology and Pathology, Institute of Animal Reproduction and Food Research, Polish Academy of Sciences, Tuwima 10, 10-748 Olsztyn, Poland

**Keywords:** Cow, Lysophosphatidic acid, Oocyte, In vitro maturation, Cumulus cells, Embryo, Cumulus expansion, Glucose metabolism, Apoptosis, Blastocyst competence

## Abstract

**Background:**

In the cow, lysophosphatidic acid (LPA) acts as an auto-/paracrine factor, through its receptors LPAR1-4, on oocytes and cumulus cells during *in vitro* maturation (IVM). The aim of the present work was to determine the effect of LPA during IVM of bovine oocytes on: 1) oocyte maturation; 2) apoptosis of COCs; 3) expression of genes involved in developmental competence and apoptosis in bovine oocytes and subsequent blastocysts; 4) cumulus expansion and expression of genes involved in the ovulatory cascade in cumulus cells; 5) glucose metabolism and expression of genes involved in glucose utilization in cumulus cells; 6) cleavage and blastocyst rates on Day 2 and Day 7 of *in vitro* culture, respectively.

**Methods:**

Cumulus-oocyte complexes (COCs) were matured *in vitro* in the presence or absence of LPA (10^−5^M) for 24h. Following maturation, we determined: oocyte maturation stage, cumulus expansion, COCs apoptosis and glucose and lactate levels in the maturation medium. Moreover, COCs were either used for gene expression analysis or fertilized *in vitro*. The embryos were cultured until Day 7 to assess cleavage and blastocyst rates. Oocytes, cumulus cells and blastocysts were used for gene expression analysis.

**Results:**

Supplementation of the maturation medium with LPA enhanced oocyte maturation rates and stimulated the expression of developmental competence-related factors (*OCT4, SOX2, IGF2R*) in oocytes and subsequent blastocysts. Moreover, LPA reduced the occurrence of apoptosis in COCs and promoted an antiapoptotic balance in the transcription of genes involved in apoptosis (*BAX* and *BCL2*) either in oocytes or blastocysts. LPA increased glucose uptake by COCs *via* augmentation of *GLUT1* expression in cumulus cells as well as stimulating lactate production *via* the enhancement of *PFKP* expression in cumulus cells. LPA did not affect cumulus expansion as visually assessed, however, it stimulated upstream genes of cumulus expansion cascade, *AREG* and *EREG*.

**Conclusions:**

Supplementation of the maturation medium with LPA improves oocyte maturation rates, decreases extent of apoptosis in COCs and sustains the expression of developmental competence related factors during oocyte maturation and subsequently affects gene expression profile at the blastocyst stage. We also demonstrate that LPA directs glucose metabolism toward the glycolytic pathway during IVM.

## Background

*In vitro* maturation (IVM) of cumulus–oocyte complexes (COCs) is a crucial part of *in vitro* embryo production, which in turn is one of the most fundamental applied techniques among the range of assisted reproductive technologies (ART) in cattle [1]. The production of competent oocytes during IVM is important for cattle reproduction concerning the ability to increase production of valuable, healthy offspring, application of nuclear transfer and transgenic technologies for agricultural usage. Although in the cow the IVM success rate is relatively high, as measured by the proportion of germinal vesicle (GV) stage oocytes that reach metaphase II (MII) stage, still only 30–40 % of *in vitro* fertilized bovine oocytes reach the blastocyst stage [2]. Oocyte maturation represents one of the key steps that limit the *in vitro* production of viable embryos [1]. Moreover, developmental competence of bovine oocytes is determined by IVM culture conditions [3, 4]. In spite of very large numbers of studies aimed at improving IVM protocols, there is still no general consensus defining the optimal maturation medium for bovine oocyte IVM; thus, continued efforts are still needed.

Lysophosphatidic acid (LPA), the simplest transmembrane phospholipid, has been regarded as an important signaling molecule participating in the regulation of reproductive functions in women [5, 6], rodents [7, 8] and farm animals including ruminants [9, 10]. Our previous studies showed that LPA is locally produced and acts in the bovine uterus [10, 11] and ovary [12, 13]. In *in vitro* studies, we found that granulosa cells were able to synthesize LPA and that it stimulated estradiol (E_2_) synthesis [13]. Moreover, we documented that LPA exerted both autocrine and paracrine effects, through its receptors LPAR1-4, on the oocytes and cumulus cells [14]. Our recently published data demonstrated that bovine embryos from Days 5 and 8 of *in vitro* culture were the site of LPA synthesis and also the target for LPA action [15].

The quantity and quality of oocytes available for *in vitro* procedures directly determines the yield and quality of *in vitro* produced blastocysts [2]. In the literature, numerous candidate genes have been described, whose level of expression in oocytes or early stage embryos is directly associated with their developmental competence. Among these genes are transcription factors, such as octamer-binding transcription factor 4 (*OCT4*) and sex-determining region Y-box 2 (*SOX2*), whose level of expression is associated with pluripotency, cell differentiation and the regulation of early embryonic development [16, 17]. There is also evidence that the mRNA abundance of receptors for insulin-like growth factors (IGF1R and IGF2R) correlates with improved morphology and growth potential of embryos [18, 19]. Another developmentally important gene is placenta-specific 8 (*PLAC8*), which was up-regulated in hatched compared to early blastocysts [20]. Additionally, BCL2 family members may be also considered as good markers of developmental potential [21].

It is well known that during IVM, cumulus cells play an essential role in proper oocyte maturation and acquisition of further developmental competence [22]. During development of bovine ovarian follicles, cumulus cells undergo expansion induced by the preovulatory luteinizing hormone (LH) surge [23, 24]. Luteinizing hormone activates intracellular signaling cascades regulating the expression of genes from the epidermal growth factor (EGF)-like family. Amphiregulin (*AREG*), epiregulin (*EREG*) and betacellulin (*BTC*) belong to this family and are critical for cumulus expansion [25, 26]. The EGF-like factors in cumulus cells act *via* the EGF receptor (EGFR) and mediate the influence of LH on expression of genes required for cumulus expansion, including prostaglandin (PG)-endoperoxide synthase 2 (*PTGS2*), tumor necrosis factor alpha-induced protein 6 (*TNFAIP6* (*TSG6*)), pentraxin 3 (*PTX3*) and hyaluronan synthase 2 (*HAS2*) [26–28]. Cumulus expansion depends on the synthesis of extracellular matrix (ECM), with hyaluronic acid as its major component. Hyaluronic acid is synthetized by HAS2 from various compounds including glucosamine and glucose [29–31]. Glucose is metabolized to hyaluronic acid by cumulus cells *via* the hexosamine biosynthetic pathway, with a key role of glutamine-fructose-6-phosphate transaminases (GFPTs) [29, 32, 33]. Cumulus cells utilize glucose also *via* glycolysis to pyruvate or lactate [34], substrates that oocytes can then use for energy production [35]. The main enzymes regulating this pathway are phosphofructokinase (PFK) and lactate dehydrogenase (LDH) [34, 35].

The present study was designed to test following hypotheses: 1) supplementation of the maturation medium with LPA influences on cumulus expansion, glucose metabolism and apoptosis in the bovine COCs, 2) supplementation of the maturation medium with LPA sustains the expression of developmental competence related factors in oocytes and subsequently affects the gene expression profile at the blastocyst stage. To test these hypotheses, we determined the effect of LPA during IVM on oocyte maturation, apoptosis of COCs and the expression of genes involved in developmental competence (*OCT4, SOX2, IGF1R*, *IGF2R* and *PLAC8*) and apoptosis (*BCL2*, *BAX* and *BAX/BCL2* ratio) in bovine oocytes and in blastocysts subsequent to fertilization. Then, we examined the influence of LPA on cumulus expansion and the expression of genes involved in the ovulatory cascade required for expansion (*AREG*, *EREG*, *BTC*, *EGFR*, *ADAM10*, *ADAM17*, *HAS2*, *PTX3*, *TNFAIP6* and *PTGS2*). We also studied the role of LPA during IVM on glucose metabolism and expression of genes involved in glucose metabolism (*GLUT1*, *GLUT4, GFPT1*, *GFPT2*, *PFKP* and *LDHA*) in cumulus cells. Finally, we evaluated the effect of LPA supplementation of the oocyte maturation medium on cleavage and blastocyst rates on Day 2 and Day 7 of *in vitro* culture of bovine embryos, respectively.

## Methods

### Experimental materials

All experimental procedures were approved by the Local Animal Care and Use Committee in Olsztyn, Poland (Agreement No. 79/2008/N, approved 19/11/2008).

### Chemicals and suppliers

Culture media for *in vitro* production of bovine embryos were purchased from Minitube (Germany). All reagents and supplements for *in vitro* culture were procured from Sigma Aldrich (Germany) unless otherwise stated. Plastic dishes, four-well plates and tubes were obtained from Nunc (Thermo Scientific, Denmark). All chemicals for reverse transcription were acquired from Invitrogen (Life Technologies, USA).

### Oocyte and cumulus cell collections

Ovaries were collected from slaughtered cows and transported to the laboratory in sterile PBS at 37 °C. Cumulus-oocyte complexes were obtained by aspiration from subordinate ovarian follicles, less than 5 mm in diameter. By assessment under a stereo microscope (Discovery V20, Zeiss, Poland; SZX7, Olympus, Poland), only COCs consisting of oocytes with homogeneous ooplasm without dark spots and surrounded by at least three layers of compact cumulus cells were selected for the study. COCs were washed two times in wash medium (M199; #M5017) supplemented with 20 mM HEPES (#H3784), 25 mM sodium bicarbonate (#S4019), 0.4 % bovine serum albumin (BSA; #A9418) and 40 μg/ml gentamicin (#G1272), and subsequently washed in maturation medium.

### *In vitro* embryo production

Groups of 25 immature COCs were placed into four-well plates (#144444) containing 400 μl of maturation medium (TCM 199 Maturation Medium (19990/0010) supplemented with 0.02 IU/ml pregnant mare’s serum gonadotropin (PMSG, #G4527), 0.01 IU/ml human chorionic gonadotropin (hCG, #C0684) and 5 % fetal bovine serum (FBS, #12106C)) overlaid with 400 μl mineral oil (#M5310), and incubated at 39 °C in a 5 % CO_2_ humidified air atmosphere for 24 h for IVM. After IVM, COCs were processed for total RNA extraction or fertilized *in vitro* and cultured. Thereafter, COCs were washed in fertilization medium (TL fertilization medium (19990/0030) supplemented with 10 μg/ml heparin (#08BK0110, WZF Polfa S. A., Poland), 20 mM sodium pyruvate (#P3662) and 0.6 % BSA). For *in vitro* fertilization, frozen-thawed semen from the same bull was used throughout the experiment. After thawing, semen was layered underneath capacitation medium (TL sperm capacitation medium (19990/0020) supplemented with 1 mM sodium pyruvate, 0.6 % BSA and 0.1 mg/ml gentamicin) and incubated for 1 h at 39 °C in a 5 % CO_2_ and humidified air atmosphere to allow recovery of motile sperm using a swim-up procedure. After incubation, the upper two-thirds of the capacitation medium were recovered, centrifuged at 200 × g for 10 min, the supernatant removed, and the sperm pellet diluted in an appropriate volume of fertilization medium to give a final concentration of 10^6^ motile sperm/ml. Groups of 25 COCs were co-incubated with spermatozoa in four-well dishes containing 400 μl of fertilization medium under 400 μl of mineral oil for 24 h at 39 °C in a 5 % CO_2_ humidified air atmosphere. The day of *in vitro* insemination was considered as Day 0. At 48 h post-insemination (hpi) embryos were separated from cumulus cells by vortexing and washed three times in wash medium. The cleavage rates were assessed and embryos with four or more cells were placed in four-well dishes containing 400 μl culture medium (SOF; synthetic oviduct fluid medium (19990/0040) supplemented with amino acids: 10 μl/ml BME (#B6766) and 20 μl/ml MEM (#M7145), 3.3 mM sodium pyruvate and 5 % FBS under 400 μl of mineral oil. Culture was carried out at 39 °C in an atmosphere of 5 % CO_2_, 5 % O_2_, 95 % N_2_ with high humidity for 7 days.

### LPA stimulation of cumulus-oocyte complexes

48 groups of 25 COCs each were used to establish all experimental groups: 1) control, oocytes matured *in vitro* for 24 h in maturation medium exposed to vehicle (PBS), and 2) LPA, oocytes matured *in vitro* in maturation medium supplemented with an LPA agonist (1-oleoyl-2-hydroxy-sn-glycero-3-phosphate sodium salt, #857130, Avanti Polar Lipids, Instruchemie, The Netherlands) at a concentration of 10^−5^M in PBS. The dose of LPA was chosen based on earlier reports in humans and rodents as well as our previous studies [6–8, 14]. Following maturation, 3 pools of the COCs from each experimental group were used for RNA isolation and 5 pools for detection of apoptosis. To assess maturation ratio at the end of the maturation time, the oocytes from another 5 pools from each experimental group were mechanically separated from cumulus cells. The denuded oocytes were observed at × 400 magnification under an inverted phase contrast microscope (CKX41, Olympus). The oocytes characterized by extrusion of the first polar body without germinal vesicle in the cytoplasm were defined as mature (MII), whereas the oocytes which had a germinal vesicle or without germinal vesicle with non-extrusion of the first polar body were defined as immature. Another 11 pools of COCs from each experimental group were fertilized *in vitro* and embryos were cultured until Day 7. Oocytes, cumulus cells and blastocysts from Day 7 of culture were used for gene expression analysis. The numbers and morphological quality of blastocysts were determined on Day 7 post-insemination. Blastocyst quality was graded according to classical morphological criteria of International Embryo Transfer Society (IETS), where grades 1–4 represent excellent, good, medium and poor embryo quality, respectively [36]. The rates of development to the blastocyst stage were calculated based on the total number of matured oocytes. The experiment was repeated ten times in batches of 25 COCs.

### Sample collection for RNA isolation and reverse transcription

For mRNA expression analysis, total RNA was extracted from oocytes, cumulus cells and blastocysts. After 24h of *in vitro* maturation, the oocytes from 5 pools of each experimental group (control or LPA) were separated from cumulus cells by vortexing. Each pool consisted of 10 denuded oocytes and all cumulus cells separated from the respective oocytes. Total RNA was extracted from 5 pools of 4 expanded blastocysts from Day 7 of culture according to the maturation groups. The oocytes, cumulus cells and blastocysts were suspended in Extraction Buffer and processed for RNA isolation according to the manufacturer’s instructions (#KIT0204, Arcturus PicoPure RNA Isolation Kit, Applied Biosystems, Life Technologies, USA). DNase treatment was performed for removal of genomic DNA contamination using RNase-free DNase Set (#79254, Qiagen, Germany). Samples were stored at −80 °C until reverse transcription. Reverse transcription (RT) was carried out using oligo (dT) 12-18 primers (#18418-012) by Super Script III reverse transcriptase (#18080-044) in a total volume of 20 μl to prime the RT reaction and produce cDNA. The RT reaction was carried out at 65 °C for 5 min then 42 °C for 60 min followed by a denaturation step at 70 °C for 15 min. RNase H (#18021-071) was used to degrade the RNA strand of an RNA-DNA hybrid (37 °C for 20 min). RT products were diluted four times and were stored at −20 °C until real-time PCR amplification.

### Quantitative real-time PCR

The quantification of mRNA for the studied genes was conducted by real-time PCR using specific primers for *OCT4*, *SOX2*, *IGF1R*, *IGF2R*, *PLAC8, BCL2*, *BAX*, *AREG*, *EREG*, *BTC*, *EGFR*, *ADAM10*, *ADAM17*, *HAS2*, *PTX3*, *TNFAIP6*, *PTGS2*, *GFPT1*, *GFPT2*, *GLUT1*, *GLUT4*, *LDHA* and *PFKP*. The results of mRNA expression were normalized to glyceraldehyde-3-phosphate dehydrogenase (*GAPDH*, an internal control) mRNA expression and were expressed as arbitrary units. This housekeeping gene was chosen using the NormFinder software, comparing three candidate genes: *GAPDH*, *β-actin* and *H2A.1* [37]. The primers were designed using an online software package (http://bioinfo.ut.ee/primer3/). Primer sequences and the sizes of the amplified fragments of all transcripts are shown in Table 1. Real-time PCR was performed with an ABI Prism 7900 (Applied Biosystems, Life Technologies, USA) sequence detection system using Maxima® SYBR Green/ROX qPCR Master Mix (# K0222, Fermentas, Thermo Scientific, USA). The PCR reactions were performed in 384-well plates. Each PCR reaction well (10 μl) contained 3 μl of RT product, 5 μM each of forward and reverse primers and 5 μl SYBR Green PCR master mix. In each reaction, we used a quantity of cDNA equivalent to 0.375 oocyte or cumulus cells from each COC and 0.15 blastocyst. Real time PCR was performed under the following conditions: 95 °C for 10 min, followed by 40 cycles of 94 °C for 15 s and 60 °C for 60 s. Subsequently, in each PCR reaction melting curves were obtained to ensure single product amplification. In order to exclude the possibility of genomic DNA contamination in the RNA samples, the reactions were also performed either with blank-only buffer samples or in the absence of the reverse transcriptase enzyme. The specificity of PCR products for all examined genes was confirmed by gel electrophoresis and by sequencing. The efficiency range for the target and the internal control amplifications was between 95 and 100 %. For relative quantification of mRNA expression levels, the real-time PCR Miner algorithm was used [38].

**Table 1:** Primers used for real-time PCR

Gene	Primer sequence (5′–3′)	Fragment size, bp	GenBank accession no.
*OCT4*	GAGAAAGACGTGGTCCGAGTG	101	NM_174580.2
	GACCCAGCAGCCTCAAAATC		
*SOX2*	TGGATCGGCCAGAAGAGGAG	89	NM_001105463.2
	CAGGCGAAGAATAATTTGGGGG		
*IGF1R*	GAGTGGAGAAATCTGCGGG	110	NM_001244612.1
	AAATGAGCAGGATGTGGAGGT		
*IGF2R*	ACCTCCGATCCTCAATCCCA	82	NM_174352.2
	TGTAGTTGAAGTGCCGGTCC		
*PLAC8*	TTTACCGCTCTGTGCCCTTT	95	NM_001025325.2
	CCATGTGAACTTGACCAAGCAT		
*BCL2*	GAGTTCGGAGGGGTCATGTG	203	NM_001166486.1
	GCCTTCAGAGACAGCCAGGA		
*BAX*	GTGCCCGAGTTGATCAGGAC	126	NM_173894.1
	CCATGTGGGTGTCCCAAAGT		
*AREG*	CTTTCGTCTCTGCCATGACCTT	100	NM_001099092.1
	CGTTCTTCAGCGACACCTTCA		
*EREG*	TCACCGCGAGAAGGATGGAG	73	XM_002688367.3
	GTACTGAAGACCAGGACGAGC		
*BTC*	GCCCCAAGCAGTACAAGCAT	100	NM_173896.2|
	GCCCCAGCATAGCCTTCATC		
*EGFR*	AAAGTTTGCCAAGGGACAAG	253	XM_002696890.2
	AAAGCACATTTCCTCGGATG		
*ADAM10*	GCTGGGAGGTCAGTATGGAA	105	NM_174496.2
	CTTTTGGCACGCTGGTGTTT		
*ADAM17*	TGTTCCACCCCAGTAACGTC	73	XM_002691486.3
	GGAAAGGGCTTGATGATGCG		
*HAS2*	CGGGGGAGATGTCCAGATTTT	120	NM_174079.2
	TGGACACATCCGAAATAAGACTG		
*PTX3*	GCCGGCAGGTTGTGAAACA	93	NM_001076259.2
	GTCTCGAGTTTCATTGGTGTCA		
*TNFAIP6*	TGCTACAACCCACATGCAAA	83	NM_001007813.2
	TCATTTGGGAAGCCTGGAGA		
*PTGS2*	TGGGTGTGAAAGGGAGGAAA	127	NM_174445.2
	AAGTGCTGGGCAAAGAATGC		
*GFPT1*	AAACACAGTCGGCAGTTCCA	80	NM_001109961.1
	TGGCTACACCAATCTCAGGC		
*GFPT2*	GAGATGTGCGGAATCTTTGCC	120	NM_001076883.1
	ACCTGCTGAGTCATAGCCTCT		
*GLUT1*	GATCCACAGAGCGCAGCC	90	NM_174602.2
	TGTCAGCTTCTTGCTGGTGG		
*GLUT4*	ATTGTGGCCATCTTTGGCTTCGTG	160	NM_174604.1
	AACCCATGCCGATGATGAAGTTGC		
*LDHA*	TCTGGATTCAGCTCGCTTCCGTTA	147	NM_174099.2
	TTCTTCAGGGAGACACCAGCAACA		
*PFKP*	TCAGAGAACCGTGCCTGGAAGAAA	112	NM_001193220.1
	TGACCACAAGCTCCTTGATCTGCT		
*GAPDH*	CACCCTCAAGATTGTCAGCA	103	NM_001034034.2
	GGTCATAAGTCCCTCCACGA		

### Detection of apoptosis in COCs

The terminal-uridine nick-end labeling (TUNEL) to detect apoptotic cells in the COCs was performed using the In Situ Cell Death Detection Kit, Fluorescein (#11684795910, Roche, Germany). At the end of the maturation time, COCs were fixed in 4 % paraformaldehyde in PBS for 1 h at room temperature (RT). Next, COCs were permeabilized in 0.3 % Triton X-100 (#T9284, Sigma Aldrich, Germany) in 0.1 % sodium citrate for 2 h at RT and washed twice in PBS. Before TUNEL labeling, positive control COCs were treated with 3000U/ml DNase (#79254, Qiagen, Germany) in the reaction buffer and incubated at RT for 1 h to induce DNA strand breaks. Following incubation, positive control and sample COCs were placed in 50 μl of TUNEL reaction mixture with the enzyme (terminal deoxynucleotidyl transferase) and incubated at 37 °C for 1 h in the dark. Negative control COCs were incubated in TUNEL label solution without the enzyme. Following incubation, COCs were washed three times in PBS, then washed and mounted in Vectashield with DAPI (Vector Laboratories, USA) on glass slides. COCs were observed under a fluorescence microscope using the DAPI filter to estimate the total number of nuclei and FITC filter for TUNEL positive cells assessment. The data was calculated as a percentage of FITC positive (apoptotic) cells within all detected DAPI positive cells. This experiment was repeated five times with 25 COCs per treatment group.

### Cumulus expansion

After IVM, COCs were visually classified into one of three groups based on the degree of cumulus expansion. Each COC was given a score from 1 to 3, where 1 designates poor expansion or lack of expansion, 2 represents partial expansion and 3 indicates complete or nearly complete expansion, as described before [39]. Cumulus expansion was expressed as a percentage of total COCs.

### Glucose metabolism

After 24 h of IVM with and without LPA, the oocyte maturation medium was recovered and stored at −80 °C until measurement of glucose and lactate concentrations. These levels were determined using an ABL 800 FLEX analyzer (Radiometer Medical, Denmark) from five experimental replicates. To determine glucose uptake, the measured glucose concentration was subtracted from the concentration of glucose in media blanks (medium cultured without cells). To determine lactate production the measured concentration in media blanks was subtracted from the concentration of the studied factors in experimental media. Glucose uptake and lactate production were expressed as mg/dL per COC and mmol/L per COC, respectively.

### Statistical analysis

The effects of treatment with LPA on gene expressions, apoptosis as well as glucose and lactate levels were tested by Student’s t-test for independent pairs. Two-way ANOVA followed by the Tukey multiple comparison test was used to determine differences in cumulus cell expansion. Maturation and blastocyst rates as well as quality of blastocysts were analyzed by Fisher’s exact test. The analyses were performed using the statistical software GraphPad PRISM 6.0 (GraphPad Software, Inc., La Jolla, CA, USA) and the results are presented as the mean ± SEM. Differences were considered statistically significant at the 95 % confidence level (*P* < 0.05).

## Results

### Effect of LPA supplementation of oocyte maturation medium on maturation rates

As presented in the Table 2, the maturation rates of oocytes matured in the presence of LPA were significantly higher than those of control oocytes (79.2 % vs. 64.9 %, respectively; *P* < 0.05).

**Table 2:** The effect of LPA supplementation of in vitro oocyte maturation medium on maturation of bovine oocytes

Supplement	Number of oocytes	Immature oocytes, n	Mature (MII) oocytes, n	Maturation rate, %	*P* value of maturation rate
*Control (PBS)*	114	40	74^a^	64,9^a^	0,0242
*LPA (10* ^*−5*^ *M)*	106	22	84^b^	79,2^b^	

Proportion of mature oocytes relative to the total number of oocytes

Different letters indicate significant differences (*P= 0,0242*), as determined by Fisher’s exact test

### Effect of LPA supplementation of oocyte maturation medium on transcription levels of genes involved in developmental competence and apoptosis in oocytes and blastocysts

**Fig. 1:**
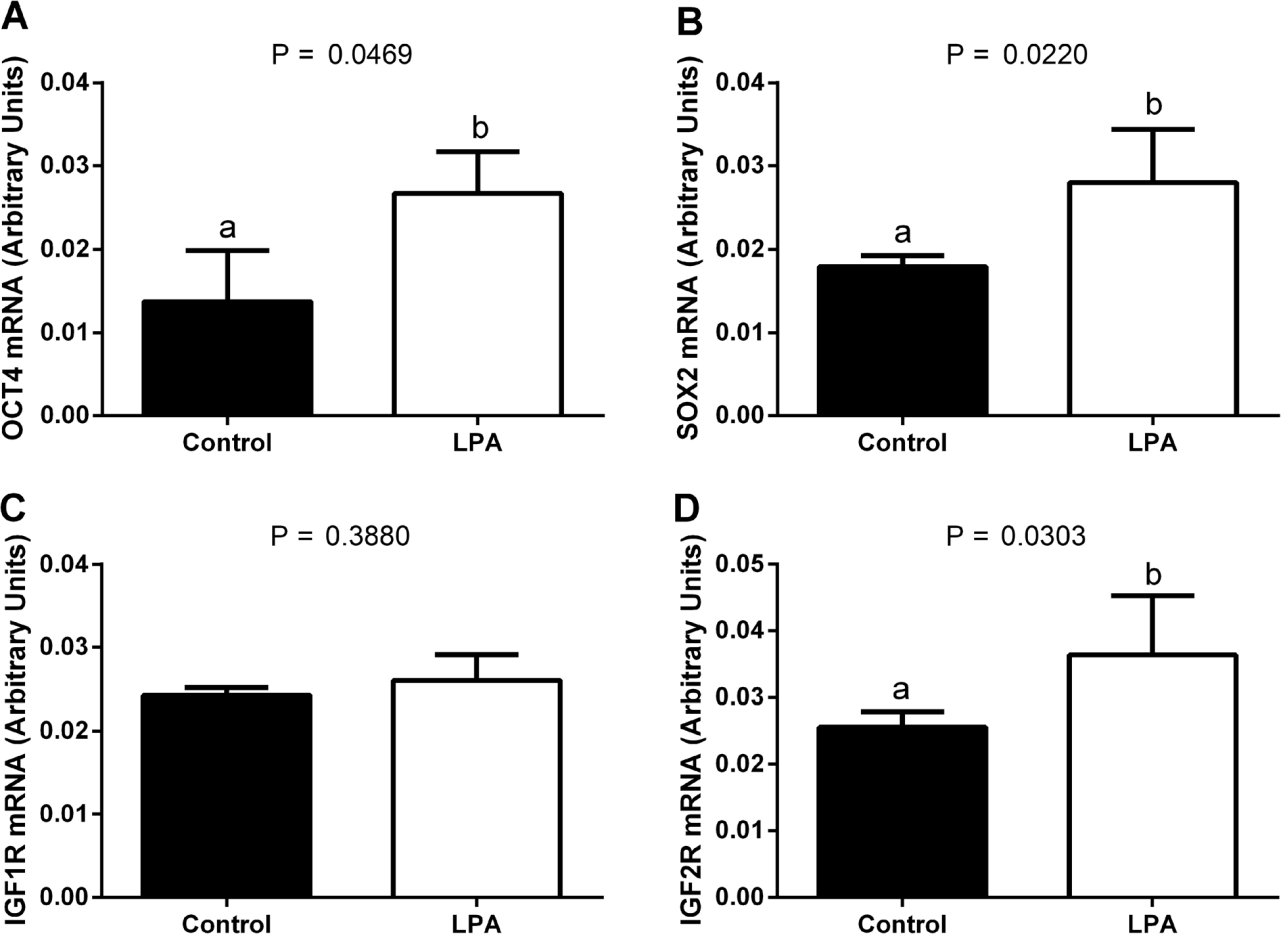
The effect of LPA (10^−5^M) supplementation of oocyte maturation medium on mRNA abundance of factors involved in developmental competence: **a** *OCT4*, **b** *SOX2*, **c** *IGF1R* and **d** *IGF2R* in oocytes. The values are presented as arbitrary units and expressed as mean ± SEM. Different letters indicate significant differences (*P* < 0.05), as determined by Student’s t-test

**Fig. 2:**
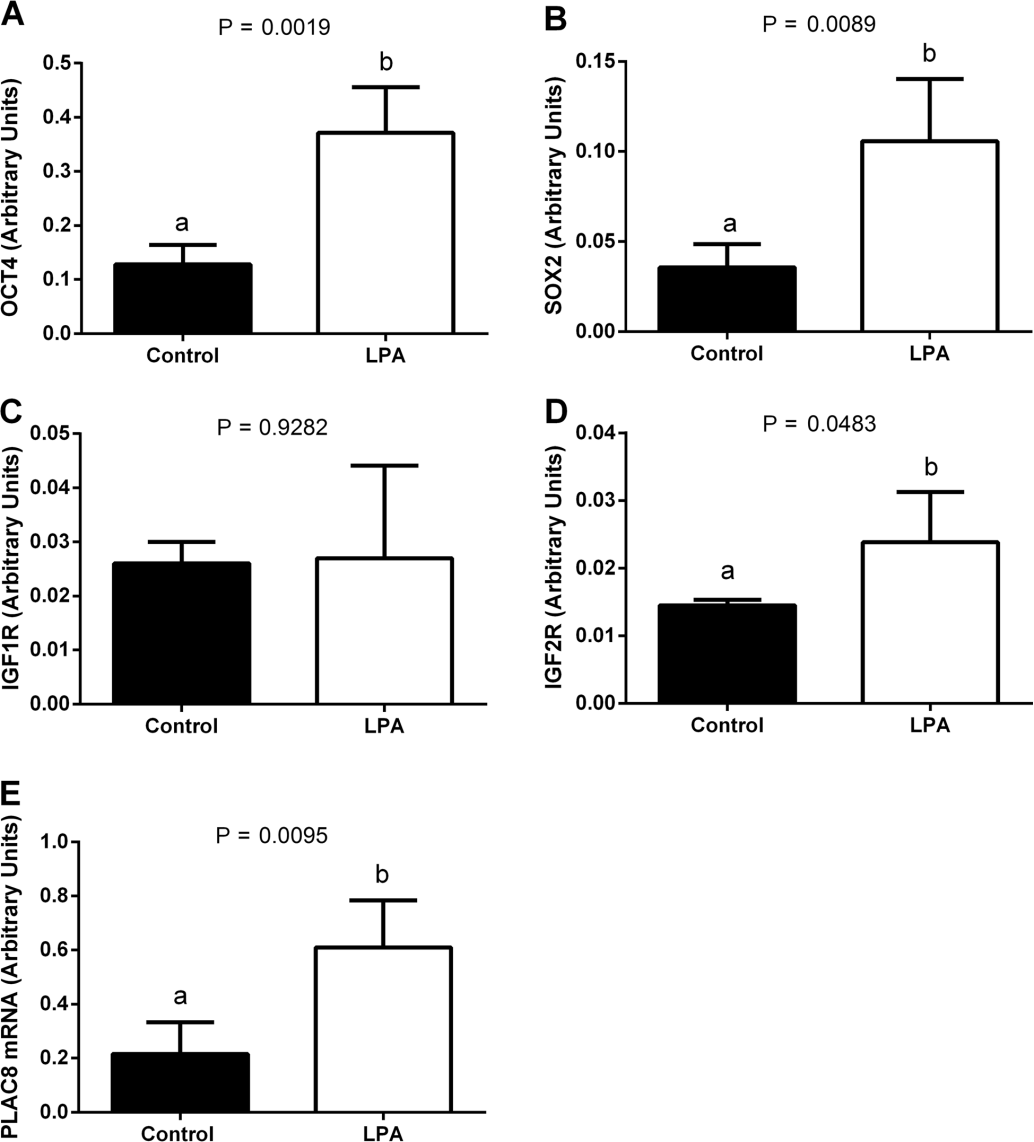
The effect of LPA (10^−5^M) supplementation of oocyte maturation medium on mRNA abundance of factors involved in developmental competence: **a** *OCT4*, **b** *SOX2*, **c** *IGF1R*, **d** *IGF2R* and **e** *PLAC8* in blastocysts. The values are presented as arbitrary units and expressed as mean ± SEM. Different letters indicate significant differences (*P* < 0.05), as determined by Student’s t-test

**Fig. 3:**
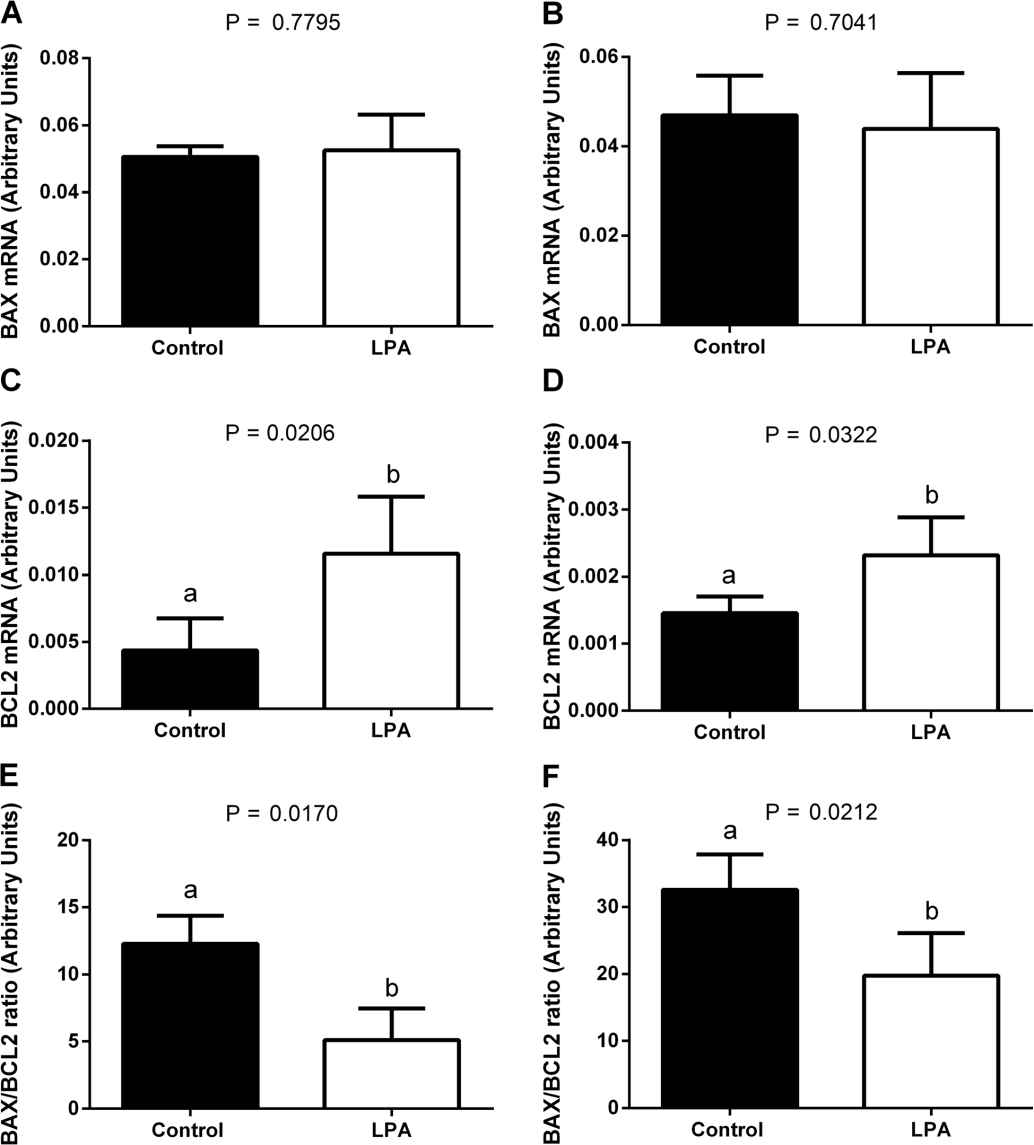
The effect of LPA (10^−5^M) supplementation of oocyte maturation medium on mRNA abundance of factors involved in apoptosis: **a**, **b** *BAX*, **c**, **d** *BCL2* and **e**, **f** the *BAX/BCL2* ratio in oocytes (**a**, **c**, **e**) and in blastocysts (**b**, **d**, **f**). The values are presented as arbitrary units and expressed as mean ± SEM. Different letters indicate significant differences (*P* < 0.05), as determined by Student’s t-test

We found higher mRNA abundance of *OCT4, SOX2* and *IGF2R* in the oocytes and blastocysts from the LPA-stimulated group compared to oocytes and blastocysts from the control group (Fig. 1a, b, d and Fig. 2a, b, d, respectively; *P* < 0.05). Supplementation of the maturation medium with LPA did not significantly influence *IGF1R* mRNA levels in the oocytes or blastocysts (Fig. 1c and Fig. 2c, respectively; *P* > 0.05). In blastocysts, there was higher mRNA abundance of *PLAC8* from the LPA-stimulated group compared to blastocysts from the control group (Fig. 2e; *P* < 0.05). We demonstrated a higher *BCL2* mRNA level and a lower *BAX/BCL2* ratio in oocytes and blastocysts from the LPA-stimulated group compared to controls (Fig. 3b, c and e, f, respectively; *P* < 0.05), whereas transcription levels of BAX were similar in the LPA-stimulated and control oocytes and blastocysts (Fig. 3a and d, respectively; *P* > 0.05).

### Effect of LPA supplementation of oocyte maturation medium on apoptosis in the COCs

**Fig. 4:**
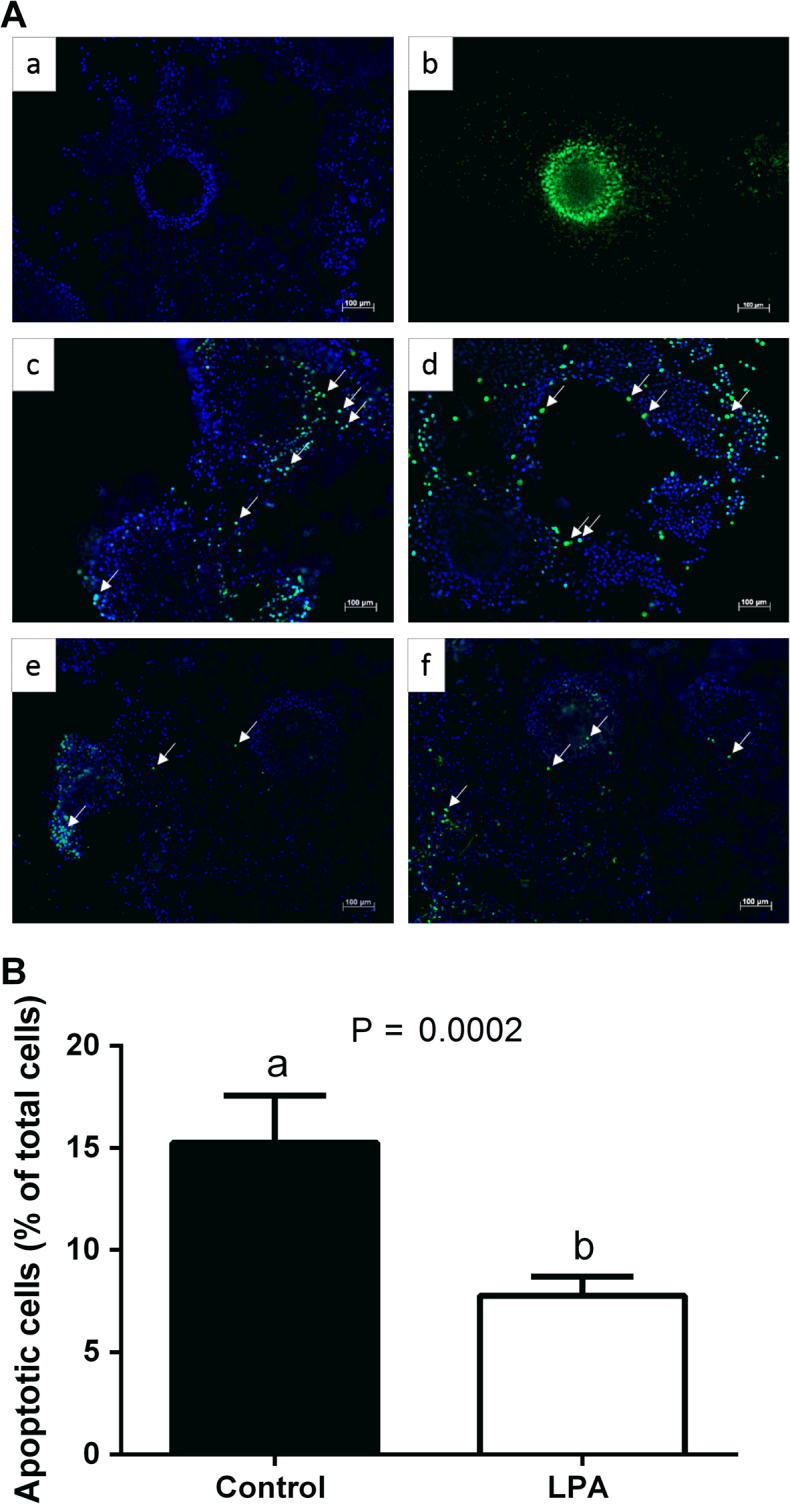
The effect of LPA (10^−5^M) supplementation of oocyte maturation medium on apoptosis in COCs. Panel **a** depicts representative fluorescent images of bovine COCs used to TUNEL labeling: a negative control, b positive control, c, d control COCs, e, f COCs matured in the presence of LPA. White arrows indicate TUNEL stained apoptotic nuclei (green) in contrast to DAPI stained nuclei (blue). Bars = 100 μm. Panel **b** depicts quantitative analysis of LPA effect on apoptosis in COCs. The data are presented as a percentage of TUNEL positive apoptotic cells within all detected DAPI positive cells and expressed as mean ± SEM. Different letters indicate significant differences (*P* <0.05), as determined by Student’s t-test

Figure 4a demonstrated representative fluorescent images of bovine COCs used to TUNEL labeling, with DNA fragmentation depicted by green fluorescence and total cells by blue fluorescence. The COCs matured in the presence of LPA showed significantly reduced proportion of TUNEL positive apoptotic cumulus cells relative to controls (Fig. 4b, 15.3 % vs. 7.8 %, respectively; *P* < 0.05).

### Effect of LPA supplementation of oocyte maturation medium on cumulus expansion and transcription levels of cumulus expansion-related genes

**Fig. 5:**
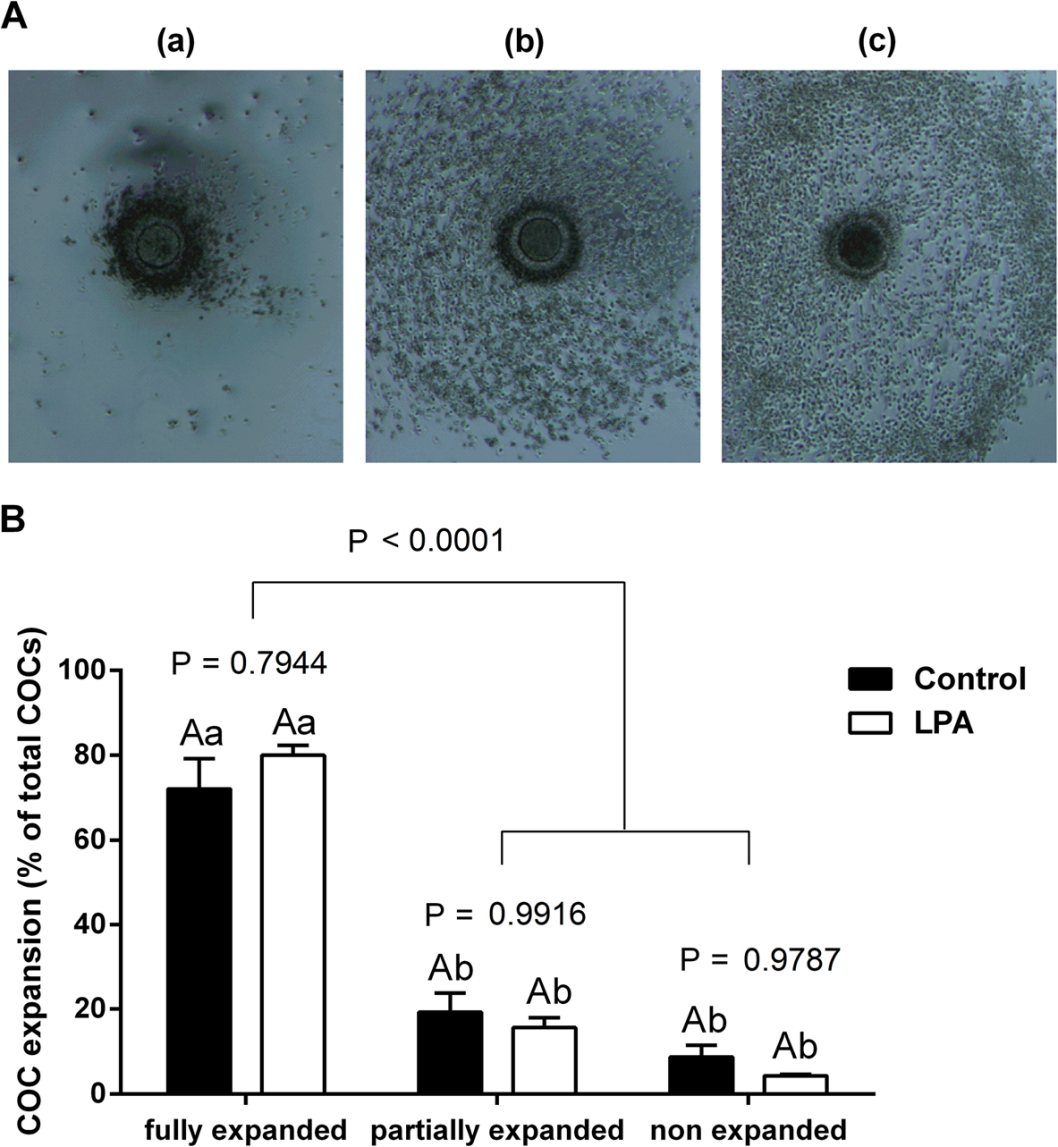
The effect of LPA (10^−5^M) supplementation of oocyte maturation medium on cumulus expansion. Panel **a** depicts representative images of bovine COCs exhibiting three degrees of cumulus expansion: **a** grade 1 (non-expanded), **b** grade 2 (partially expanded) and **c** grade 3 (fully expanded). Original magnification × 50. Panel **b** depicts quantitative analysis of LPA effect on cumulus expansion. The values are presented as percentage of COCs and expressed as mean ± SEM. Capital letters indicate statistical significance (*P* > 0.05) between two treatments whilst different small letters indicate significant differences (*P* < 0.05) within each treatment, as determined by two-way ANOVA followed by the Tukey multiple comparison test

**Fig. 6:**
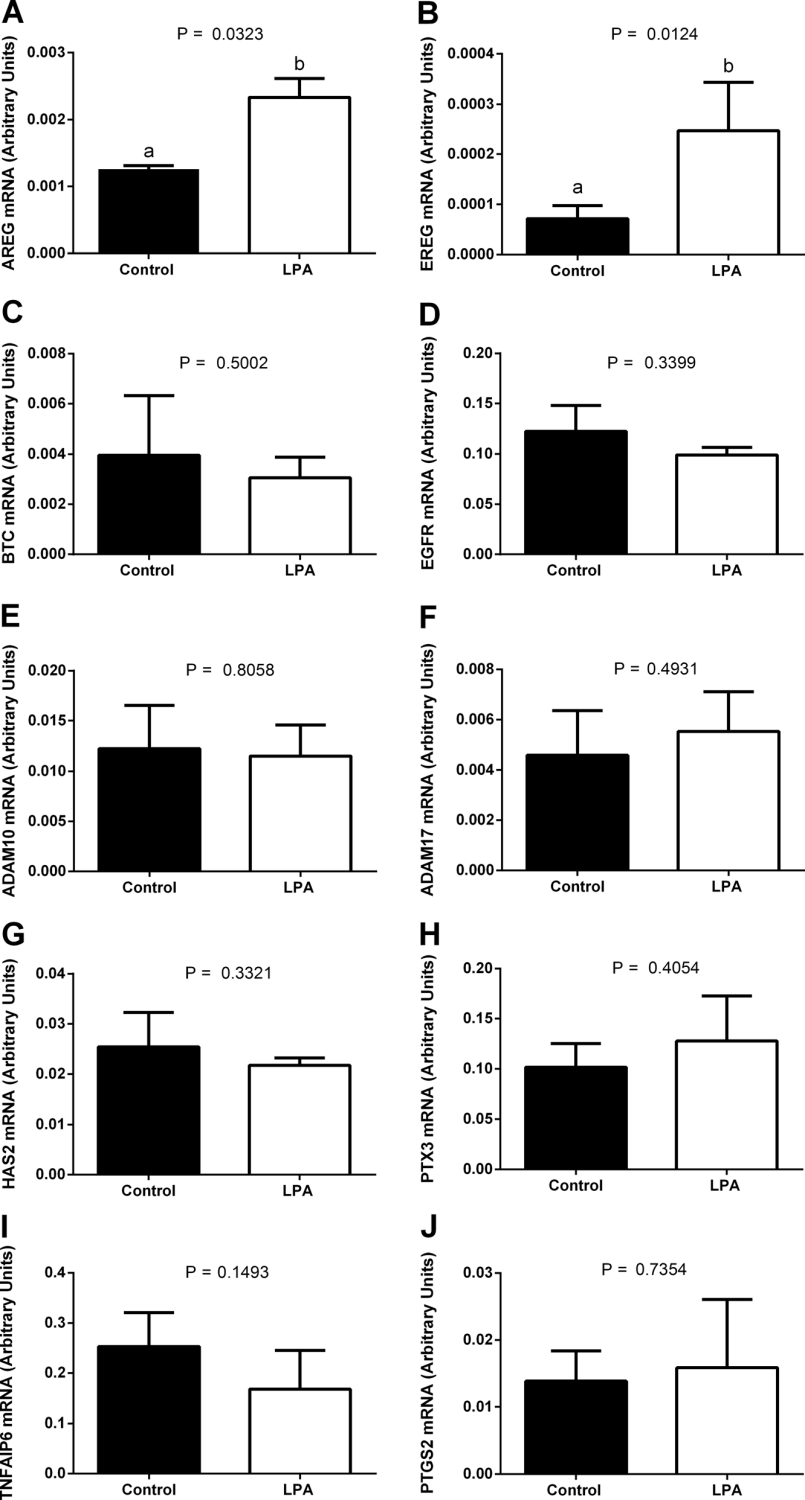
The effect of LPA (10^−5^M) supplementation of maturation medium on mRNA abundance of cumulus expansion-related factors: **a** *AREG*, **b** *EREG*, **c** *BTC*, **d** *EGFR*, **e** *ADAM10*, **f** *ADAM17*, **g** *HAS2*, **h** *PTX3*, **i** *TNFAIP6* and **j** *PTGS2* in cumulus cells. The values are presented as arbitrary units and expressed as mean ± SEM. Different letters indicate significant differences (*P* < 0.05), as determined by Student’s t-test

The percentages of grade 1 (non-expanded), 2 (partially expanded) and 3 (fully expanded) COCs were all similar in COCs from the LPA-stimulated group compared to COCs from the control group (Fig. 5; *P* > 0.05). We found higher mRNA abundance of *AREG* and *EREG* in cumulus cells from the LPA-stimulated group compared to those from the control group (Fig. 6a, b; *P* < 0.05). The expression levels of *BTC, EGFR, ADAM10, ADAM17, HAS2, PTX3, TNFAIP6* and *PTGS2* in cumulus cells did not significantly differ between groups (Fig. 6c–j; *P* > 0.05).

### Effects of LPA supplementation of oocyte maturation medium on glucose metabolism and transcription levels of genes involved in glucose metabolism

**Fig. 7:**
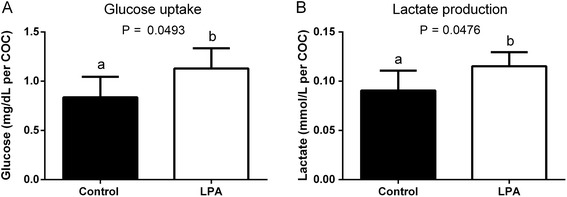
The effect of LPA (10^−5^M) supplementation of oocyte maturation medium on (**a**) glucose uptake and (**b**) lactate production by COCs. Glucose uptake and lactate production are expressed as mean ± SEM and presented as mg/dL per COC and mmol/L per COC, respectively. Different letters indicate significant differences (*P* < 0.05), as determined by Student’s t-test

**Fig. 8:**
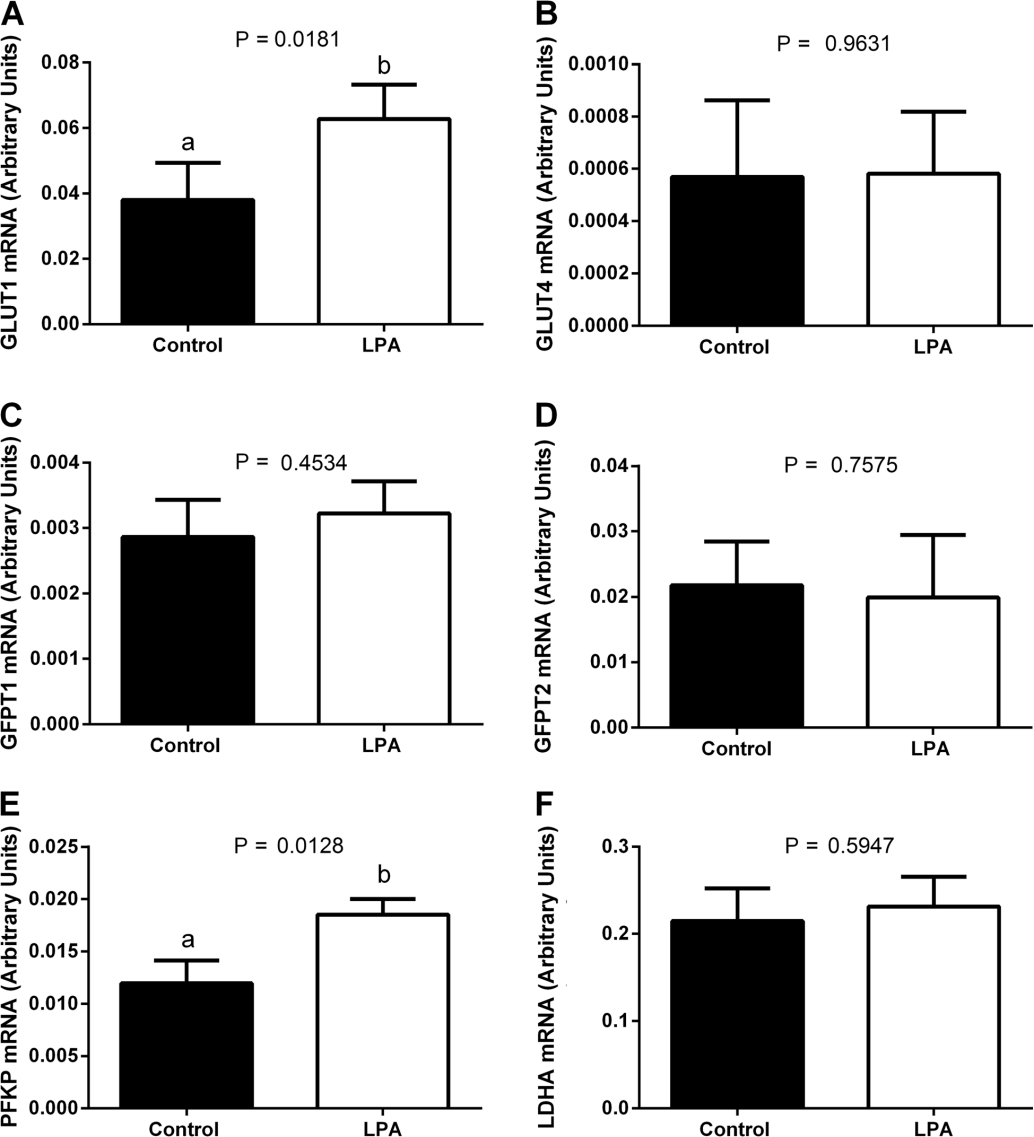
The effect of LPA (10^−5^M) supplementation of maturation medium on mRNA abundance of genes involved in glucose metabolism: **a** *GLUT1*, **b** *GLUT4*, **c** *GFPT1*, **d** *GFPT2*, **e** *PFKP* and **f** *LDHA* in cumulus cells. The values are presented as arbitrary units and expressed as mean ± SEM. Different letters indicate significant differences (*P* < 0.05), as determined by Student’s t-test

LPA stimulated glucose uptake and lactate production by COCs (Fig. 7a, b; *P* < 0.05). We found that LPA increased the abundance of mRNA encoding *GLUT1* and *PFKP* in cumulus cells compared to the control group (Fig. 8a, e; *P* < 0.05). Addition of LPA to the maturation medium had no effect on *GLUT4, GFPT1*, *GFPT2* or *LDHA* mRNA levels (Fig. 8b–d, f; *P* > 0.05).

### Effect of LPA supplementation of oocyte maturation medium on embryonic development and blastocyst morphological quality

As shown in Table 3, we did not find any significant differences in the cleavage rates on Day 2 between the control group and the LPA-stimulated group (57.6 % vs. 55.1 %, respectively; *P* > 0.05). The blastocyst rates on Day 7 were similar in the control group and the LPA-stimulated group (25.0 % vs. 28.7 %, respectively; *P* > 0.05). The proportions of quality grade 1 and grade 2 Day 7 blastocysts were also similar in the LPA and control groups (63.4 % vs. 58.1 %, respectively; *P* > 0.05).

**Table 3:** The effect of LPA supplementation of in vitro oocyte maturation medium on the bovine embryo development and blastocyst morphological quality

Supplement	Matured oocytes, n	Cleaved embryos, n (%)	*P* value of cleavage rate	Blastocyst on Day 7, n (%)	*P* value of blastocyst rate	Qualities 1 and 2, n (%)	*P* value of blastocyst qualities
*Control (PBS)*	248	143 (57,6)	0,5871	62 (25,0)	0,3628	36 (58,1)	0,5946
*LPA (10* ^*−5*^ *M)*	247	136 (55,1)		71 (28,7)		45 (63,4)	

Proportion of the cleaved embryos on Day 2 and blastocysts on Day 7 of embryo culture relative to the total number of matured oocytes

Proportion of qualities 1 and 2 of blastocysts relative to the total number of blastocysts

*P* values determined by Fisher’s exact test

## Discussion

The present study demonstrated that LPA supplementation of the maturation medium *in vitro* enhanced the maturation rates of the oocytes. Similarly, the maturation rates of the oocytes cultured in the presence of LPA were improved in mice [8, 40], golden hamsters [7] and pigs [41]. We found also the stimulatory effect of LPA on the expression of genes involved in developmental competence in bovine oocytes and in the subsequent blastocysts. Previously published evidence showed that IVM conditions alter gene expression patterns in bovine oocytes [42]. Moreover, in our previous studies, LPA supplementation of oocyte maturation medium increased expression of the quality marker genes: follistatin (*FST*) and growth and differentiation factor 9 (*GDF9*) in the oocyte [14]. The oocyte maturation environment affects not only gene expression in oocytes, but also affects blastocyst development and transcript abundance in bovine embryos [4, 43]. Here, supplementation of the oocyte maturation medium with LPA increased *OCT4* and *SOX2* mRNA abundance in both oocytes and blastocysts. These factors are involved in transcriptional regulation during early embryonic development and cell differentiation [16, 17]. In mice, *OCT4-*deficient 1-cell embryos are arrested at multi-cell stages, which suggests that *OCT4* is required for early embryo development prior to formation of the blastocyst [44]. Similarly, embryos with depleted *SOX2* failed to develop into blastocysts [17]. It has also been shown that transcripts of *OCT4* and *SOX2* are present in oocytes and early bovine embryos [45]. Gendelman and Roth [46] documented higher mRNA levels of *OCT4* in mature oocytes collected from cows in the cold season than in those from the hot season. Transcription of *OCT4* was also increased in early- vs. late-cleaved embryos [47]. The authors also documented higher *OCT4* transcript levels in early-cleaved embryos developed in the cold season compared with embryos from the hot season [47]. These results suggest that seasonally induced alterations in *OCT4* expression are involved in the reduced developmental competence noted for oocytes and embryos obtained in the hot season [46, 47]. Knockdown of *SOX2* in bovine zygotes reduced blastocyst rates, probably because of a failure in maternal-embryonic genome transition [48]. In the present study, LPA stimulated mRNA expression of *OCT4* and *SOX2*, indicating a supporting role of LPA in the pluripotency pathway, beginning during oocyte maturation and subsequently affecting gene expression profiles at the blastocyst stage. However, in our previous studies, supplementation of the culture medium with LPA between Days 2 and 8 had no effect on *OCT4* blastocyst transcription [15].

The insulin-like growth factors exert an important role during early embryo development *via* binding to their receptors (IGF1R and IGF2R) on the target cells [18, 49]. The expression patterns of bovine *IGF1R* and *IGF2R* showed increased mRNA levels during oocyte maturation, which then decreased gradually until embryonic genome activation at the 8- to 16-cell stage and increased again to reach the highest levels in hatched blastocyst [50]. This transcriptional profile accounts for the important role of *IGF1R* and *IGF2R* during IVM and then again, after hatching, in formation of the filamentous conceptus [50]. Liu et al. [19] showed that the transcript level of genes coding *IGF1R* and *IGF2R* correlated with morphological assessment and growth potential of human embryos. Therefore, the mRNA expression patterns of *IGF1R* and *IGF2R* represent good markers of embryo quality [19]. In our studies, we found a stimulatory effect of LPA during IVM on *IGF2R* transcription in oocytes and then subsequently in blastocysts. Moreover, these results support our previous data, that supplementation of the embryo culture medium with LPA enhanced mRNA expression of *IGF2R* in bovine blastocysts [15].

In the present study, we also demonstrated that the mRNA transcription level of *PLAC8* in blastocysts was augmented by LPA during IVM. *PLAC8*, which is a placental-enriched gene, was found to be upregulated in the bovine endometrium during the preimplantation stage of early pregnancy compared to the respective period of the estrous cycle [51]. It was also reported that, in epithelial cells of the bovine endometrium, transcription of *PLAC8* is induced by interferon-tau (IFNτ), which in ruminants is the main embryonic signal for pregnancy recognition [52]. Enhanced expression levels of *PLAC8* were also demonstrated in expanded and hatched blastocysts compared to early blastocysts, which suggested its important role in embryo apposition and embryo-maternal interaction taking place before the blastocyst escapes its zona pellucida [20]. El-Sayed et al. [53] showed significantly higher expression of *PLAC8* in *in vitro*-derived blastocysts that lead to calf delivery compared to blastocysts that were resorbed. Similarly, in *in vivo* studies, *PLAC8* was highly expressed in embryo biopsies that led to calf delivery compared to biopsies of blastocysts that failed to establish or maintain pregnancy [54]. Taken together, data for *PLAC8* showed the same expression tendency for blastocysts selected based on the pregnancy outcome after transferring in *in vivo*- and *in vitro*-produced embryos [53, 54]. Accordingly, the expression profile of *PLAC8* revealed an association with developmental competence of bovine embryos, independently of their environmental origin [54]. Taking all the above into consideration, LPA is able to influence on bovine oocyte maturation leading to the enhanced oocyte maturation and to the increased expression of developmentally important genes in the oocytes and subsequently at the blastocyst stage. These effects, although they were not translated into the enhanced *in vitro* blastocyst quality, may be of relevance for subsequent *in vivo* embryo competence.

Apoptosis is a physiological process occurring during preimplantation embryo development either *in vivo* or *in vitro* [55]. Nevertheless, a higher level of apoptosis was observed in blastocysts derived *in vitro* than in their *in vivo* counterparts [55]. Numerous studies reporting the relationship between the occurrence of apoptosis in oocytes and blastocysts and their developmental competence appear to be contradictory [21, 56–59]. In fact, in some studies, developmentally more competent oocytes exhibited early signs of atresia [56]. In contrast, other studies reported that the degree of apoptosis in COCs was negatively correlated with the developmental competence of the oocyte [58]. The present study demonstrated that the exposure to LPA during oocyte maturation reduced the number of TUNEL-positive, apoptotic nuclei in bovine COCs. Similarly, supplementation of the culture medium with LPA reduced the occurrence of apoptosis in the porcine blastocyst [41]. According to Yang & Rajamahendran [21], the BAX to BCL2 protein ratio was associated with the quality of oocytes and embryos. It was also shown that a higher incidence of apoptosis in late cleaving, and thus less viable, embryos in comparison to early cleaving ones resulted in decreased developmental competence [60]. There is also evidence in the literature that the expression of genes related to apoptosis such as *BCL2* and *BAX* was altered during oocyte and embryo culture [14, 15, 57]. Therefore, the expression of these genes may be an indicator related to culture conditions and also serve as a marker of oocyte and blastocyst viability [57, 59]. Melka et al. [59] demonstrated that gene expression of *BAX* was increased in morphologically poor quality embryos compared with those exhibiting good developmental competence. In our previous studies, supplementation of oocyte maturation medium with LPA decreased oocyte *BAX* mRNA abundance and increased oocyte *BCL2* transcript levels, as well as reducing the oocyte *BAX* to *BCL2* ratio [14]. Moreover, supplementation of the culture medium with LPA induced a decrease in blastocyst transcription of *BAX* and an increase in transcription of *BCL2* [15]. Similarly, the treatment of porcine embryos with LPA resulted in the increased expression of antiapoptotic *BCL2L1* gene and in the decreased expression of the proapoptotic *BAX* and caspase 3 (*CASP3*) genes [41]. In this study, we found that LPA added to the oocyte maturation medium enhanced *BCL2* mRNA levels and decreased the *BAX* to *BCL2* ratio in oocytes and blastocysts from the LPA-stimulated group, whereas LPA had no effect on transcription levels of *BAX* either in oocytes or blastocysts. There are some discrepancies between our present and previous data [14, 15] regarding the influence of LPA on mRNA expression in bovine oocytes. This may be due to differences in composition of oocyte maturation media or to variations in the type of LPA agonists that were used in these studies. The differences concerning *BAX* mRNA levels in blastocysts can arise from LPA supplementation of different kinds of media for *in vitro* production of bovine embryos: oocyte maturation or embryo culture medium. Nevertheless, supplementation with LPA, during both oocyte maturation and embryo culture, triggered an anti-apoptotic gene expression profile both in oocytes and blastocysts, which may be reflected in oocyte and blastocyst viability. Moreover, LPA decreased the extent of apoptosis in the COCs. Thus, we suggested that although LPA treatment did not affect embryo development until Day 7 of *in vitro* culture, the influence on the apoptosis may be relevant for subsequent *in vivo* embryo survival.

The role of cumulus cells is crucial for maturation of oocytes and their developmental competence [22], since removal of cumulus cells during IVM significantly affects subsequent embryo development [61, 62]. Many studies have focused on gene expression in oocytes in order to find specific molecular markers to characterize oocyte quality [63, 64]. Nevertheless, the gene expression profile in cumulus cells seems to be as important as gene expression patterns in oocytes, in view of the supporting role of cumulus cells in acquisition of oocyte competence [24, 65, 66]. In our previous studies, we demonstrated that in bovine cumulus cells, LPA added to the oocyte maturation medium reduced the expression of cysteine proteinases, i.e., the cathepsins CTSB, CTSK, CTSS and CTSZ [14], higher expression of which was associated with developmental incompetence of bovine oocytes [65]. The gene expression profile in cumulus cells can also contribute towards establishing a non-invasive approach for assessment of oocyte quality in order to predict further developmental potential [24, 66, 67]. Routinely, in ART, developmentally competent oocytes are selected based on the number and compactness of the surrounding cumulus cells [63, 65, 68]. Moreover, oocytes associated with cumulus cell investments that fail to expand are unable to ovulate and/or be fertilized [69]. Thus far, a number of genes essential for cumulus expansion have been identified, including factors from the EGF-like family, *AREG, EREG, BTC* [25, 26, 70], their receptor *EGFR* [25, 26], disintegrin and metalloproteinase family members *ADAM10, ADAM17* [70] as well as factors involved in ECM formation, *PTGS2*, *TNFAIP6*, *PTX3* and *HAS2* [26–28]. In the preovulatory ovarian follicle, the LH surge triggers a cascade of events, including oocyte meiotic maturation and cumulus expansion [23, 24]. However, evidence in rodents suggests that many LH effects are indirect, for example it was found that LH-induced resumption of oocyte meiosis was mediated by AREG and EREG [25, 26]. In the present study, we found that LPA increased the abundance of mRNA encoding *AREG* and *EREG*. These two EGF-like factors are considered to be major stimuli for cumulus expansion and oocyte maturation [25, 26]. In mice, AREG and EREG played a pivotal role in the activation of cumulus expansion: in COCs with mural granulosa cells removed, in which LH did not affect cumulus expansion, the action of these two growth factors was even more effective in stimulating expansion than in intact COCs [25]. Following the LH surge, AREG and EREG were released from the membrane by actions of ADAM10 or ADAM17 [71, 72] and activated the EGFR in order to propagate signals throughout the follicles prior to ovulation [25]. Subsequently, the EGFR ligands induced gene expression of *PTGS2, HAS2,* and *TNFAIP6*, which is required for remodeling of the ECM during cumulus expansion [25–28]. In the cow, AREG also affected cumulus expansion by stimulating glycolysis and enhancing glucose consumption and lactate production [73]. Here, among the genes examined that are involved in bovine cumulus expansion, LPA increased mRNA expression of *AREG* and *EREG.* Although LPA did not stimulate cumulus expansion as assessed visually, it could play a supporting role during bovine oocyte IVM *via* augmentation of these two major factors for cumulus expansion. The lack in differences in cumulus expansion and at the same time enhanced expression of *AREG* and *EREG* by LPA can arise from limitation of visual evaluation of morphological changes in COCs under stereo microscope. While the increase in gene expression of *AREG* and *EREG* did not translate into bioactive protein and induction of expansion, effects of LPA on AREG- and EREG-mediated cumulus expansion cannot be excluded. Moreover, considering that LPA stimulated glycolysis in the COCs and that AREG affected bovine cumulus expansion also by stimulating glycolysis and enhancing glucose consumption and lactate production [73], we postulate that cumulus expansion can be indirectly supported by LPA-mediated increase in expression of *AREG* and *EREG* and by the enhancement of glucose metabolism in the glycolytic pathway, without differences in the morphology of COCs.

It has been also shown that some metabolic parameters of COCs can be used as predictive markers of oocyte quality [74]. Higher glucose metabolism in oocytes from adult cows *vs*. those from prepubertal cows, a comparison which represents a bovine model for investigation of poor quality oocytes, suggests that glucose utilization is positively correlated with oocyte developmental potential [74]. Glucose is an important energy substrate that can facilitate oocyte maturation, cumulus expansion and subsequent blastocyst development [75]. During IVM, cumulus cells play a pivotal role in the provision of nutrients to the oocyte. [76, 77]. In our study, addition of LPA to the oocyte maturation medium increased mRNA expression of *GLUT1*, but had no effect on *GLUT4* mRNA abundance. During IVM, GLUTs provide passive, energy-independent transport of glucose through the COCs [32]. Glucose transporter 1 is the most abundant isoform of GLUTs and its expression was detected throughout preimplantation development in cattle, from the oocyte to the elongating Day 16 conceptus [78]. The mRNA abundance of *GLUT1* was reduced in bovine blastocysts produced *in vitro* compared with *in vivo* obtained embryos, which suggests that *GLUT1* transcription relates to the developmental competence of bovine embryos [79]. Wang et al. [32] proposed the pathway of intercellular glucose transport in mouse COCs, in which glucose enters cumulus cells *via* the GLUTs system and then is transferred to the oocyte through the gap junctions. Moreover, inhibition of GLUTs leads to decreased glucose uptake in cumulus cells [32]. On the other hand, Saito et al. [76] demonstrated that in the absence of cumulus cells, glucose utilization was at a negligible level in denuded murine oocytes. Thus, cumulus cells seem to be responsible for providing rather intermediates of glucose metabolism such as pyruvate or lactate, which are preferred energy substrates for the oocyte [35]. In our study, LPA increased glucose uptake and lactate production by bovine COCs, suggesting its role in directing glucose metabolism down the glycolytic pathway. The linear relationship between glucose consumption and lactate production by bovine COCs suggests that the majority of glucose during IVM is utilized *via* glycolysis [77]. Furthermore, supplementation of the maturation medium with LPA stimulated expression of mRNA encoding *PFKP*, a key enzyme involved in the regulation of glycolysis. Cetica et al. [34] showed that the high activity of PFK in cumulus cells and its very low activity in the oocyte indicates a major participation of the glycolytic pathway in cumulus cells to supply substrates to the oocyte. Moreover, supplementation of the maturation medium with pyruvate or lactate plus NAD increased the number of denuded oocytes that matured, indicating that the oocytes used these glycolytic metabolites for energy production [35]. Due to the important role of glucose during IVM [74, 75], there are various pathways to metabolize glucose [34, 77]. Beside glycolysis, glucose is utilized *via* the hexosamine biosynthetic pathway for synthesis of ECM components, especially hyaluronic acid [29, 30, 33]. Glutamine-fructose-6-phosphate transaminases are the main enzymes regulating hexosamine biosynthesis and perform a key function in controlling the pathway metabolic rate [33]. Here, LPA did not affect either the expression of mRNA encoding *GFPT1* and *GFPT2* or the mRNA abundance of *HAS2* in the cumulus cells. The increased glucose uptake without enhanced lactate production by bovine COCs towards the end of IVM suggests possible glucose consumption for the formation of the ECM [80]. Therefore, the results of the present study showed that LPA increased glucose uptake and stimulated lactate production by bovine COCs, whereas it had no effect on the synthesis of ECM.

## Conclusions

To conclude, this study provides evidence, from both functional and gene expression studies, that allows better understanding of LPA actions in the regulation of IVM of bovine COCs. The study demonstrates that supplementation of oocyte maturation medium with LPA enhances the maturation rates of the oocytes. The exposure to LPA during oocyte maturation influences on the expression patterns of genes associated with oocyte and blastocyst competence. The addition of LPA to the maturation medium increases mRNA abundance of *OCT4*, *SOX2, IGF2R* in both oocytes and blastocysts and also mRNA expression of *PLAC8* in the blastocysts. Moreover, LPA reduces the occurrence of apoptosis in the COCs as well as promotes an anti-apoptotic balance in transcription of genes involved in apoptosis (*BCL2* and *BAX*) either in the oocytes or the blastocysts, which may be reflected in oocyte and blastocyst viability. We were also the first to demonstrate that LPA directs glucose metabolism toward the glycolytic pathway. The results of this study demonstrates that LPA increases glucose uptake by bovine COCs *via* augmentation of *GLUT1* expression in cumulus cells as well as stimulating lactate production *via* the enhancement of *PFKP* expression in cumulus cells. Although LPA did not affect cumulus expansion as visually assessed, during bovine oocyte IVM it stimulated the upstream genes of the cumulus expansion cascade: *AREG* and *EREG*.
